# Optimal Sunshade Configurations for Space-Based Geoengineering near the Sun-Earth L_1_ Point

**DOI:** 10.1371/journal.pone.0136648

**Published:** 2015-08-26

**Authors:** Joan-Pau Sánchez, Colin R. McInnes

**Affiliations:** 1 Space Research Centre, Cranfield Universiy, Cranfield, Bedfordshire, United Kingdom; 2 School of Engineering, University of Glasgow, Glasgow, United Kingdom; SETI Institute, UNITED STATES

## Abstract

Within the context of anthropogenic climate change, but also considering the Earth’s natural climate variability, this paper explores the speculative possibility of large-scale active control of the Earth’s radiative forcing. In particular, the paper revisits the concept of deploying a large sunshade or occulting disk at a static position near the Sun-Earth L_1_ Lagrange equilibrium point. Among the solar radiation management methods that have been proposed thus far, space-based concepts are generally seen as the least timely, albeit also as one of the most efficient. Large occulting structures could potentially offset all of the global mean temperature increase due to greenhouse gas emissions. This paper investigates optimal configurations of orbiting occulting disks that not only offset a global temperature increase, but also mitigate regional differences such as latitudinal and seasonal difference of monthly mean temperature. A globally resolved energy balance model is used to provide insights into the coupling between the motion of the occulting disks and the Earth’s climate. This allows us to revise previous studies, but also, for the first time, to search for families of orbits that improve the efficiency of occulting disks at offsetting climate change on both global and regional scales. Although natural orbits exist near the L_1_ equilibrium point, their period does not match that required for geoengineering purposes, thus forced orbits were designed that require small changes to the disk attitude in order to control its motion. Finally, configurations of two occulting disks are presented which provide the same shading area as previously published studies, but achieve reductions of residual latitudinal and seasonal temperature changes.

## Introduction

With the increasing recognition that climate change is happening, together with seemingly weak efforts to sufficiently reduce greenhouse gas (GHG) emissions to avoid dangerous climate change [1], a number of methods that seek to counteract the adverse climatic effects of GHG emissions have been proposed and investigated [2]. Research into these alternatives to counteract anthropogenic climate change is still controversial, but is generally regarded as prudent by the scientific community [2, 3]. These deliberate interventions in the Earth’s climate system are generally known as geoengineering [4].

Geoengineering methods are divided into those that aim to remove CO_2_ from the atmosphere, referred as carbon dioxide removal methods (CDR), and those that aim to manage radiative forcing in order to compensate for GHG emissions, referred to as solar radiation management (SRM). CDR methods tackle the principal problem of climate change, although the removal of CO_2_ is a slow process and thus these methods typically require a long duration to impact on the climate system. On the other hand, SRM can rapidly reduce radiative forcing and thus is seen as a possible last resort for the mitigation of the adverse effects of anthropogenic-driven climate change.

SRM methods aim to compensate for GHG-induced warming by reducing either the incidence or the absorption of solar radiation. This can be achieved by a range of approaches, such as enhancing surface albedo (e.g., white roofs or more reflective crop varieties) or enhancing atmospheric albedo (e.g., cloud seeding or stratospheric aerosols) [2]. Space-based methods, instead, aim to divert incoming solar radiation before it reaches the Earth.

Numerous space-based geoengineering methods have already been discussed in the literature. Some visionary concepts envisage, for example, an artificial Earth ring of passive scattering particles [5, 6], fabricated either on Earth or resourced from the Moon or asteroids. The use of unprocessed space resources in the form of large clouds of dust capable of scattering solar radiation [7, 8] has also been suggested as a means to reduce the engineering complexity entailed in assembling large structures in space, e.g., space sunshades [9]. Space-based proposals however are not seen as affordable and timely as compared to terrestrial geoengineering techniques, such as stratospheric aerosols [2]. Yet, they do have the fundamental advantage that they do not involve direct manipulation of either the Earth’s atmosphere or its surface properties.

**Fig 1:**
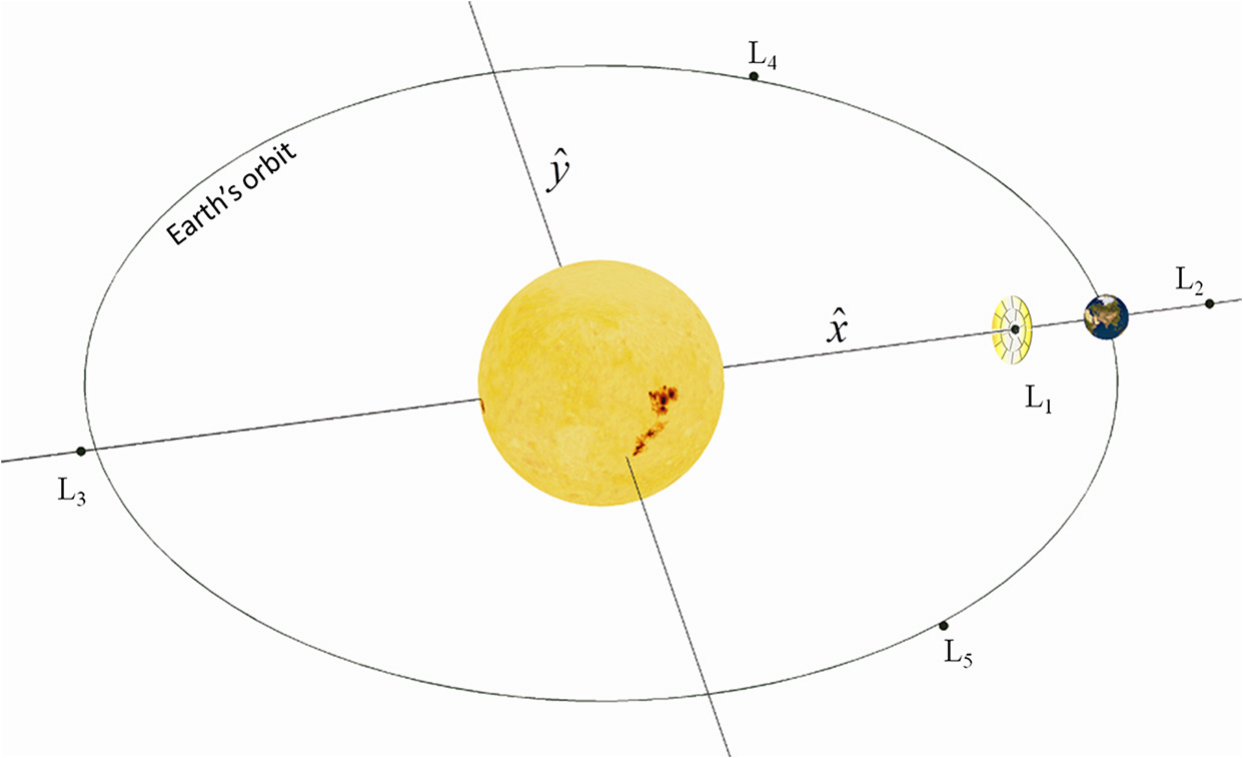
Occulting disk near the Sun-Earth Lagrange L_1_ point. The L_1_ point rotates around the Sun with the same angular speed as the Earth, thus allowing an occulting disk to remain in a position where it casts continuous shade on the Earth.

Perhaps the most practical space-based concept, yet clearly still challenging, is that of deploying a large sunshade or occulting disk, or a swarm of smaller structures covering the equivalent shading area to reduce solar insolation. Typically, these proposals require deploying the occulting structure close to the L_1_ Lagrange equilibrium point on the Sun-Earth line, as shown in Fig 1, some 1.5x10^6^ km sunward of the Earth. The estimated mass of the deployed structure is in the order of 10^7^–10^8^ tonnes [9–12]. While this is clearly a vast space-based endeavour, its mass is already at the same engineering scale as terrestrial civil engineering projects (e.g., the Chinese Three Gorges Dam). Furthermore, as discussed elsewhere [2, 13], sunshades appear to be one of the most efficient methods to tackle climate change, and also one of the few proposals with the potential to achieve the required reduction of solar insolation.

Most scenarios for space-based geoengineering target a reduction of solar insolation of 1.7% in order to offset the effects of a doubling of CO_2_ concentration. This engineered reduction of solar insolation has been shown to yield a decrease of the global mean surface temperature sufficient to compensate for the anthropogenic-driven increase [14]. Hence, the shade cast by the occulting disk must reduce the solar flux received at the Earth, S = 1367 W/m^2^ [15], by ΔS = 23.24 W/m^2^. A quick estimate of the radius of the occulting disk *R*_*Disk*_ can be obtained by comparing the solid angle subtended by the disk and the Sun, as seen from the Earth [16]. Thus,

$$R_{Disk}=R_{⊙}\frac{d_{Disk}}{d_{⊙}}\sqrt{\frac{\Delta S}{S}}$$ (1)

where *R*_⊙_ is radius of the Sun, *d*_*Disk*_ is distance of the occulting disk from the Earth, *d*_⊙_ is the distance of the Sun from the Earth and ratio of the decrease of the solar flux Δ*S*/*S* is targeted at 1.7%. At the *classical* Sun-Earth Lagrange L_1_ point, which lies at a distance from the Earth of order 1.5x10^6^ km, an occulting solar disk radius *R*_*Disk*_ of 915 km, or a structure with an equivalent area of 2.6x10^6^ km^2^ would be required. However, this location is in fact unfeasible, since the force exerted by photons impinging on the occulting disk, i.e. solar radiation pressure (SRP), moves the equilibrium position sunwards from the classical location (see Problem Statement and Methods for further discussion).

While space-based geoengineering is arguably non-invasive, it has been shown using General Circulation Models (GCM), that a uniform insolation reduction of 1.7% would drive important changes to regional climates [17], with warming at high latitudes while cooling below necessary in sub-tropical regions. These residual changes to regional climates may still cause important damage to the local ecosystems and economies [18]. Later work [19], on the other hand, has also shown that particular spatio-temporal shading patterns may potentially suppress some of these regional effects, such as warming at high latitudes, changes in tropical rain patterns or ice cover changes.

Largely motivated by this prior work, this paper is aimed at investigating the possibility of designing new configurations of orbiting sunshades with the potential of containing not only the increase in global mean temperature, but also residual regional anthropogenic climate change. This paper then focuses on the deployment of large sunshades to enable insolation control. Many other alternatives have already been suggested in the literature, as mentioned earlier. A more complete review of space-based geoengineering proposals can be found in McInnes et al. [20], where the potential for active control of solar insolation is also briefly discussed. This paper, however, considers a scenario where multiple occulting disks, or sunshades, are deployed near the Sun-Earth Lagrange L_1_ point. This work investigates the use of artificial out-of-plane equilibria and periodic motion near this equilibrium position in order to deliver efficient shading patterns capable of coping with latitudinal and seasonal changes of the Earth’s climate due to a doubling of CO_2_ concentration.

Previous work in astronautics [9–12] has already discussed the feasibility of implementing the sunshade concept to counteract the increase of global mean temperature. On the other hand, the potential for spatio-temporal shading patterns to regionally suppress anthropogenic climate variability has also been demonstrated using fully-coupled atmosphere-ocean general circulation models [19, 21]. This paper, however, seeks to investigate the possibility to engineer particular Earth shading patterns by means of designing configurations of mobile sunshades near the Sun-Earth Lagrange L_1_ point. Earth shading patterns are thus sought to mitigate not only global, but also regional effects of GHG emissions.

In order to attempt a preliminary understanding of the effects of altering the solar insolation, an appropriate climate model is required. Moreover, in order to gain an understanding of regional effects (e.g., latitudinal and seasonal variations in surface temperature), while at the same time performing a numerically intensive search for optimal sunshade configurations, an appropriately balanced level of complexity of the climate model is also needed. Hence, in this first approximation to the problem a simple, but globally resolved, energy balance model, referred to thereafter as GREB [22], is used in order to provide insight into the coupling between the dynamics of the occulting disk and the Earth’s climate.

As discussed in the original publication by Dommenget and Floter (2011), as well as briefly in the first part of the paper, GREB has been shown to be capable of simulating the main characteristics of global warming. Hence, optimal sunshade configurations are sought that return the largest possible fraction of Earth’s surface to a climate within ±0.1 C° difference of the surface temperatures of that of the 1xCO_2_ world, as computed by GREB. It is acknowledged, however, that GREB may provide only a crude representation of the climate response at regional and seasonal scales, since it neglects processes that may be important factors in regional changes. Further work must then perform the optimization with fully-coupled atmosphere-ocean general circulation models, but also discuss in more detail what the targeted *ideal* climate should be (i.e. rain patterns, ice cover, extreme events, etc.).

The remainder of the paper is divided in two main parts: Problem Statement and Methods and Results and Discussion. Within Problem Statement and Methods, section I describes the consequences of the force exerted by photons impinging on the sunshade within the dynamical framework of the Circular Restricted Three Body Problem. Section II briefly describes the climate model used, i.e. GREB, and presents both the 2xCO_2_ world scenario (baseline), which sunshades will attempt to counteract, and the control scenario (i.e. CO_2_ concentration of 340 ppm (1xCO_2_) with no artificial shading). Section III discusses the climate achieved by the classical sunshade configuration (i.e. static shade at L_1_ point). While section IV defines the geoengineering performance index used thereafter to measure how well the geoengineering climate matches the control scenario. In Results and Discussion, section I defines the multi-objective optimization problem and discusses the results for two particular cases. Section II computes the control angles (i.e. required attitude changes) that are necessary for the sunshades to satisfy the prescribed geoengineering motion, as computed in section I, and thus demonstrate than these can actually be engineered. Finally, section III engages in a more speculative discussion and reviews the validity of the results.

## Problem Statement and Methods

### SRP-displaced Sun-Earth L_1_ Point

As mentioned earlier, the preferred location for an occulting disk is at the position where the gravitational forces from the Sun and Earth, together with the solar radiation pressure (SRP), balance in a reference frame rotating along with the Earth. This location is generally known as the Sun-Earth Lagrange L1 point, and allows the occulting disk to remain in a position where it can cast continuous shade on the Earth (see Fig 1). If SRP is not considered, this equilibrium position is located at 1.5x10^6^ km from the Earth. However, the force exerted by photons impinging on the occulting disk is not negligible, and displaces the equilibrium location sunwards. This is because, as experienced by the disk, the inverse square SRP reduces the effective gravitational attraction of the Sun, displacing sunwards the point where the gravitational forces from the Sun and Earth and the force due to SRP cancel each other in the rotating reference frame.

The effect of SRP can be conveniently quantified using the ‘lightness’ parameter *β*, which is the ratio of the force due to SRP and solar gravity, and can be expressed as a function of the area-to-mass ratio of the occulting disk *A*_*d*_/*M*_*d*_ [23]:

β=σ*AdMdσ*=L⊙⋅Q2πGM⊙c≈1.53⋅Q[g/m2] (2)

where σ* is the critical loading parameter, *L*_⊙_ is the solar luminosity, *G* is the gravitational constant, *M*_⊙_ is the mass of the Sun and *c* the speed of light in a vacuum. Finally, the constant *Q* is a measure of the optical properties of the sunshade, such that *Q* = 1 is equivalent to a perfectly reflecting surface, i.e. specular reflection, and *Q* = 0 would account for photons passing through the disk without any scattering. Thus, the actual value *Q* depends on the optical properties of the occulting disk material; thus, for example, a reflecting metallic disk would have *Q*~0.91, while a non-reflective black occulting disk would be equivalent to *Q*~0.17 [11].

Assuming the motion of the occulting disk within the frame of the Circular Restricted Three-body Problem (CR3BP) [24], the acceleration on the disk due to the solar radiation pressure can be expressed as [23]:

$$a=\beta \frac{1-\mu }{r_{1}^{2}}{\left(\hat{n}\cdot {\hat{r}}_{1}\right)}^{2}\hat{n}$$ (3)

where *r*_1_ is the distance to the Sun, ${\hat{r}}_{1}$ denotes its unit vector, $\hat{n}$ is the unit vector normal to the occulting disk surface and away from the Sun ($\hat{n}\cdot {\hat{r}}_{1}>0$) and the mass parameter *μ* = *m*_2_ / (*m*_1_ + *m*_2_) assumes the mass of the primary *m*_1_ as the mass of the Sun and the mass of the secondary *m*_2_ as the mass of the Earth-Moon system, thus *μ* = 3.0401x10^–6^.

**Fig 2:**
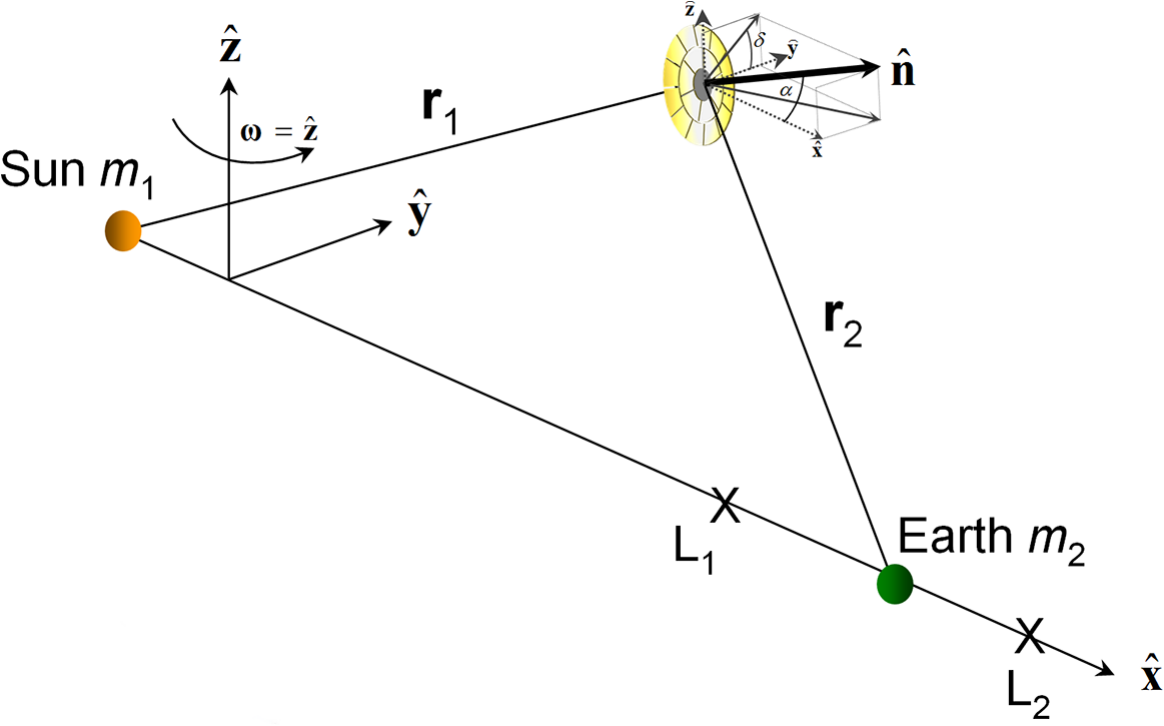
Circular restricted Three Body Problem frame of reference, vectors and occulting disk attitude definition (i.e. cone *α* and clock *δ* control angles).

In such a system, as described in Fig 2, the equations of the motion of the occulting disk can be expressed as:

$$\begin{array}{l} \ddot{x}-2\dot{y}-\frac{\partial U}{\partial x}=a_{x}\\ \ddot{y}+2\dot{x}-\frac{\partial U}{\partial y}=a_{y}\\ \ddot{z}-\frac{\partial U}{\partial z}=a_{z} \end{array}$$ (4)

where the SRP acceleration (*a*_*x*_, *a*_*y*_, *a*_*z*_) can be defined by means of the cone and clock angles (*α*,*δ*). In this work, the cone angle *α* defines the angle between the Sun-line and the normal to the surface of the occulting disk and the clock angle *δ* is defined here as the angle between the *y*-axis and the projection of the normal to the surface of the occulting disk on the *y*-*z* plane (see Fig 2). Considering both the latter definitions and Eq 4, the acceleration vector (*a*_*x*_, *a*_*y*_, *a*_*z*_) can then be expressed as:

$$\left(\begin{array}{ccc} \beta \frac{1-\mu }{r_{1}^{2}}\cos^{3}\alpha , & \beta \frac{1-\mu }{r_{1}^{2}}\cos^{2}\alpha \sin\alpha \cos\delta , & \beta \frac{1-\mu }{r_{1}^{2}}\cos^{2}\alpha \sin\alpha \sin\delta \end{array}\right)$$ (5)

The three-body gravitational potential *U* is expressed as:

$$U=\frac{1}{2}\left(x^{2}+y^{2}\right)+\frac{1-\mu }{r_{1}}+\frac{\mu }{r_{2}}$$ (6)

The occulting disk position vectors with respect to the Sun *r*_1_ and the Earth *r*_2_ are defined with respect the rotating reference frame as:

$$\begin{array}{l} r_{1}=\left[x+\mu ,y,z\right]\\ r_{2}=\left[x-\left(1-\mu \right),y,z\right] \end{array}$$ (7)

Finally, the units of the system are chosen such that *ω* = 1 and *m*_1_+*m*_2_ = 1, thus the mass parameter is defined as *μ* = *m*_2_ / (*m*_1_ + *m*_2_), and then *m*_1_ = 1-*μ* and *m*_2_ = *μ*.

The exact location of the SRP-displaced L_1_ point can then be computed by searching for the collinear equilibrium position (*x*_*e*_,0,0) such that ∇_*x*_ *U*(*x*_*e*_,0,0) + *a*_*x*_(*x*_*e*_,0,0) = 0 and (1-μ)>*x*_*e*_>-μ. At the equilibrium position, the vector normal to the occulting disk will be $\hat{n}=\left(\begin{array}{ccc} 1 & 0 & 0 \end{array}\right)$ and the Sun-line vector will be **r**_1_ = (*x*_*e*_ + *μ*, 0, 0). Then ∇_*x*_ *U*(*x*_*e*_,0,0) + *a*_*x*_(*x*_*e*_,0,0) = 0 resolves to the quintic equation [24]:

$$\gamma^{5}-\left(3-\mu \right)\gamma^{4}+\left(3-2\mu \right)\gamma^{3}+\left(1-2\mu -\left(\beta +1\right)\left(1-\mu \right)\right)\gamma^{2}+2\mu \gamma -\mu =0$$ (8)

where *γ* = *x*_*e*_ − (1 − *μ*). Thus, as shown by Eq 8, the actual equilibrium position of the occulting disk depends on the ‘lightness’ parameter *β*, which at the same time, as defined in Eq 2, depends on area-to-mass ratio of the occulting disk.

**Fig 3:**
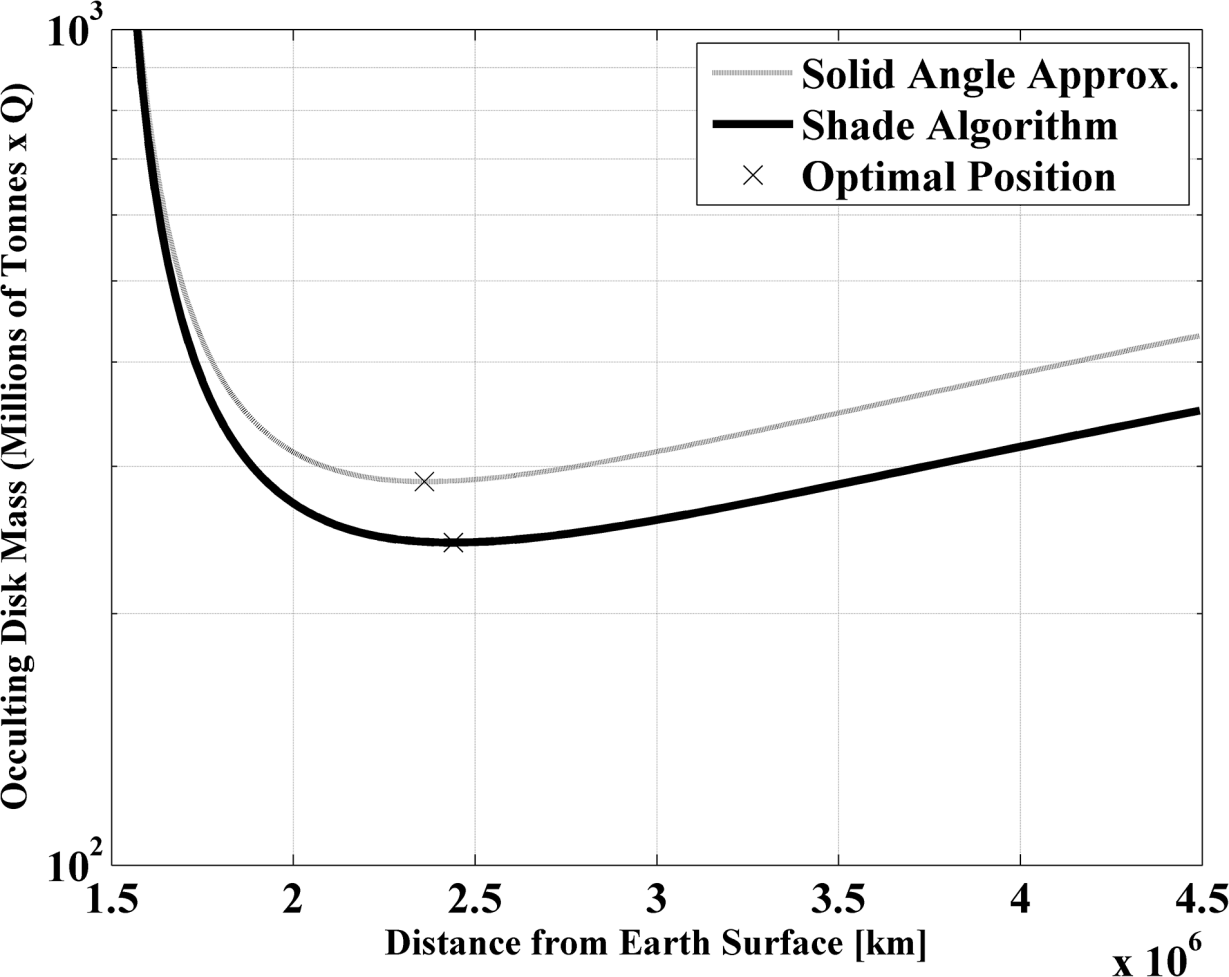
System mass required to geoengineer Earth. Classical L_1_ position≈1.5x10^6^km.

It then follows, that since the disk area required is defined as a function of the distance to the Earth, as expressed by Eq 1, the equilibrium position is therefore uniquely defined by the mass of the system. Accordingly, the mass necessary to shade the Earth by 1.7% can be expressed as a function of the distance from the Earth. Fig 3 shows firstly how as the occulting disk is located closer to the classical L_1_ position the mass of the disk grows to infinity, as more mass is required to anchor the disk at a given location by reducing the solar radiation pressure induced acceleration. Secondly, as the disk is located further from the classical L_1_ point this mass requirement eases, but the area requirement grows to maintain the required solid angle to shade the Earth, which results in an optimal mass position located close to 2.5x10^6^km sunward of the Earth.

Note that two similar, but distinct, sets of results are plotted in Fig 3. The curve referred to as *Solid Angle Approximation* is computed by means of Eq 1, as well as assuming that the entire solar disk radiates with the same intensity [11]. The curve referred to as *Shade Algorithm* contains two improvements to the latter model in order to obtain more accurate results, especially for the shade patterns cast by an occulting disk. First, the solid angle subtended by the Sun and the disk are computed numerically, as is their respective position on the celestial sphere as seen by an observer located at a given position on the surface of the Earth. Then, the circle-circle intersection formula can be used to estimate the reduction of the flux, and numerical integration over the entire Earth surface yields the average reduction of solar flux, whose results are shown in Fig 3 labelled as shade algorithm. However, the solar disk does not radiate at the same intensity, as was assumed for the solid angle approximation, but appears darker at the edges than at the centre, thus the relative position of the occulting disk with respect to the solar disk is important. This effect, known as solar limb darkening, is also modelled by the shade algorithm. Further details on the specific model used to account for the solar limb darkening are given in section III.

Note also that Fig 3 estimates the mass of the occulting disk assuming a solar radiation coefficient Q equal to 1. The figure linearly scales with Q, thus for example, as described by McInnes [11], a non-reflecting black occulting disk with Q~0.17 would result in a factor of six saving in mass, thus a minimum mass of 40 million tonnes, instead of 240 million. It is clear then that the lower the solar radiation coefficient Q, the lower the mass of the geoengineering system. Angel [12] discusses the minimum reflectivity achievable with near- to mid-term technology. In his work [12], a solution with Q~0.04 and area-to-mass ratio near 4 g/m^2^ is presented, which would result in a geoengineering structure of 14 million tonnes. Obviously, there are many options to be explored, for example, while Angel’s design requires a highly manufactured occulting disk, and thus most likely may require to be fabricated and launched from Earth, McInnes proposes to fabricate a disk from asteroid material. Despite the fact that McInnes’ [11] proposal is three times heavier than Angel’s, the launch mass required, if space in-situ manufacturing is an option, may be significantly lower [25].

The goal of the paper is however to investigate efficient configurations of orbiting sunshades. Since, as shown by Fig 3, the minimum mass of a geoengineering system occurs at 2.44x10^6^ km, this is then taken as the baseline distance to the Earth were the optimal configurations of sunshades are deployed, in the remaining of the paper. Nevertheless, technological limitations in thin film and lightweight structure fabrication may make this distance ultimately unattainable, and therefore some closer position to the Earth may be used instead [12]. In this case, similar configurations, as studied here, exist with disk area and orbital motion scaled accordingly to cast similar shade onto the Earth.

### GREB: Baseline (2xCO_2_) and Control (1xCO_2_) Scenarios

**Fig 4:**
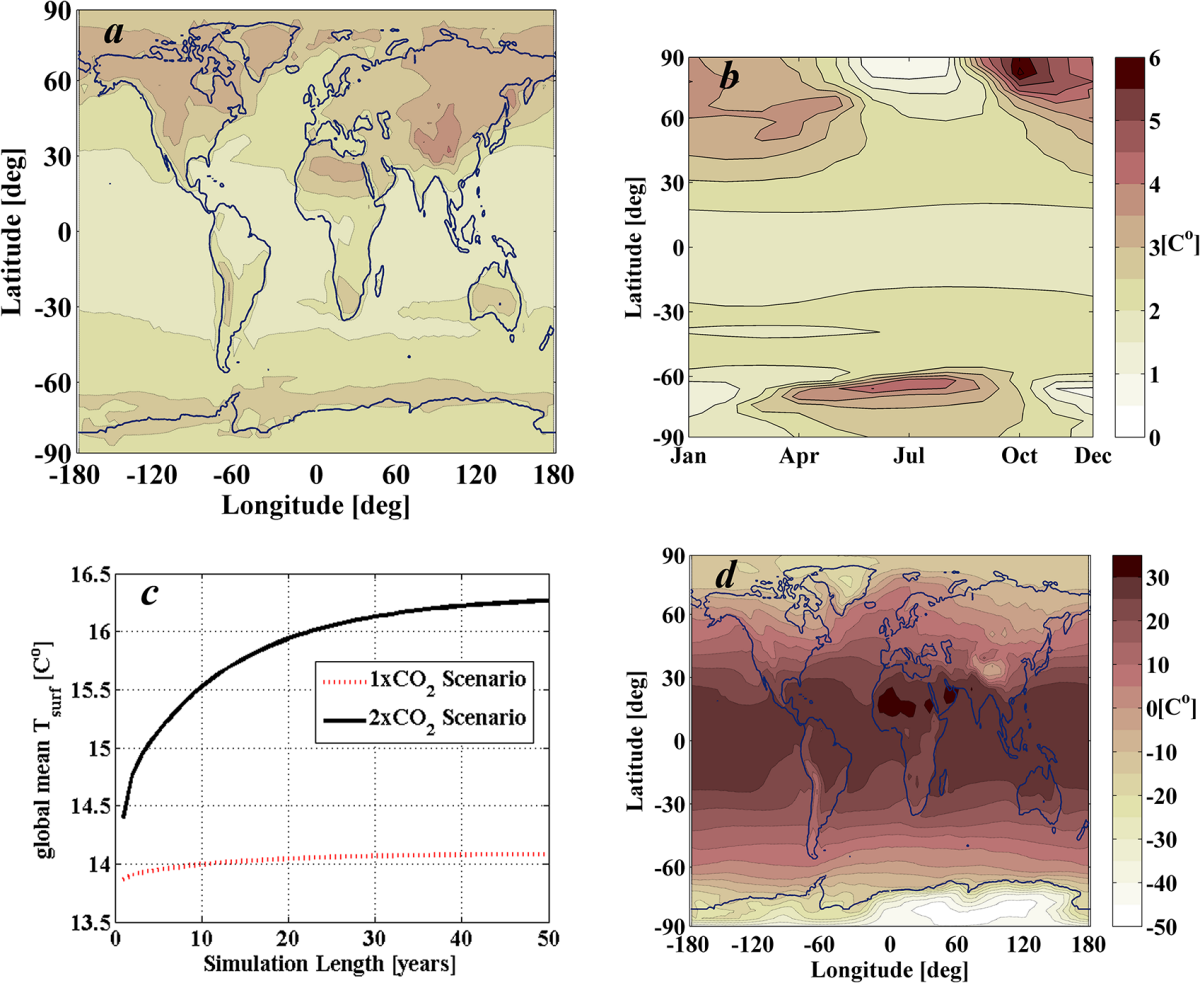
Climate response of 2xCO_2_ world scenario as computed by GREB model in a 50 year simulation. The initial CO_2_ concentration is set to 340 ppm (a level similar to that of 1980s), thus the doubled concentration is 680 ppm. *a*) Mean differences of monthly mean surface temperatures between the 2xCO_2_ scenario and 1xCO_2_ control scenario at the last year of simulation in GREB’s 3.75°x3.75° horizontal grid. *b*) Latitudinal and seasonal increase of temperature computed by averaging longitudinal data. *c*) Evolution of the global mean surface temperature T_surf_ of the two scenarios considered (1xCO_2_ and 2xCO_2_) as a function of the length of the simulation. *d*) Mean surface temperatures during the last year of simulation (50^th^ year) for the 2xCO_2_ scenario.

The GREB model is a strongly simplified climate model that captures only the main physical processes. These physical processes include solar and thermal radiation, the hydrological cycle, sensible heat, advection, diffusion, the sea ice and deep oceans. All these processes are represented also by very simplified models [22]. GREB simulates temperatures, in a 3.75°x3.75° horizontal grid, resolved in three vertical layers (atmosphere, surface and subsurface ocean), as well as the atmospheric water vapour content in the atmospheric level. Also, in contrast to GCMs, the model assumes fixed atmospheric circulation, cloud cover and soil moisture, which are given as boundary conditions. Hence, GREB does not simulate the internal chaotic climate variability associated with weather fluctuations, and as shown by Fig 4*C*, converges towards a new equilibrium solution once an external forcing, such as the increase in greenhouse gases or changes in solar insolation, is introduced in the model. These new forcing conditions are interpreted as small perturbations, such that the atmospheric and ocean circulation suffer negligible changes (i.e. boundary conditions).

GREB then provides a simple conceptual model that allows for a fast tool to simulate the climate response to changes in CO_2_ concentration and solar insolation. Fig 4 shows the climate response to a doubling of CO_2_ concentration with step change from 340 ppm (a level similar to that of 1980s) to a concentration of 680 ppm. The GREB response reaches an approximate equilibrium condition after 50 years (see Fig 4*C*), which is computed in about 15 minutes on a standard desktop computer. This set up provides the baseline scenario for global warming that the remainder of the paper will attempt to offset by optimally placing occulting disks near the Sun-Earth L_1_ equilibrium position.

Note that the baseline scenario (Fig 4) is not realistic in many senses. Firstly, because we consider a step change in the concentration of CO_2_ from 340 ppm to 680 ppm, while a more realistic scenario should consider a continuous increase in CO_2_ concentration as predicted by the scenarios issued by the Intergovernmental Panel on Climate Change (IPCC). Secondly, because GREB is a simplistic representation of the climate, and neglects some important feedbacks that may indeed be the main factors for regional changes in the real climate. Despite this, GREB provides a reasonable representation of the surface temperatures response to the global greenhouse gas increase. It simulates the global mean climate sensitivity and large-scale regional aspects of the warming pattern within the uncertainties of the IPCC-model ensemble [26].

**Fig 5:**
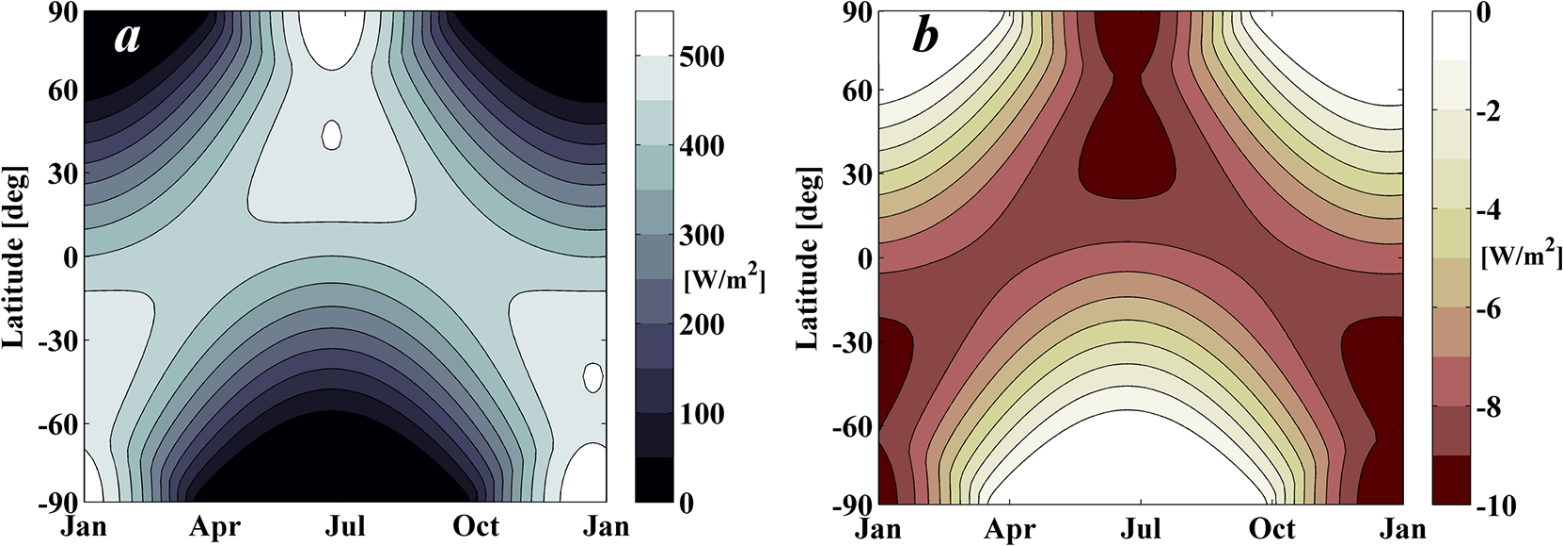
Daily-averaged latitudinal incoming solar radiation over one complete year. Note that the Earth’s orbital eccentricity is assumed zero. *a*) Natural isolation as a function of latitude and time of year. *b*) Shade cast by a 1,434 km radius occulting disk at the optimal SRP displaced L_1_ point (i.e. decrease of insolation with respect to the natural insolation). The shade is represented as daily-averaged decrease in solar insolation.

**Fig 6:**
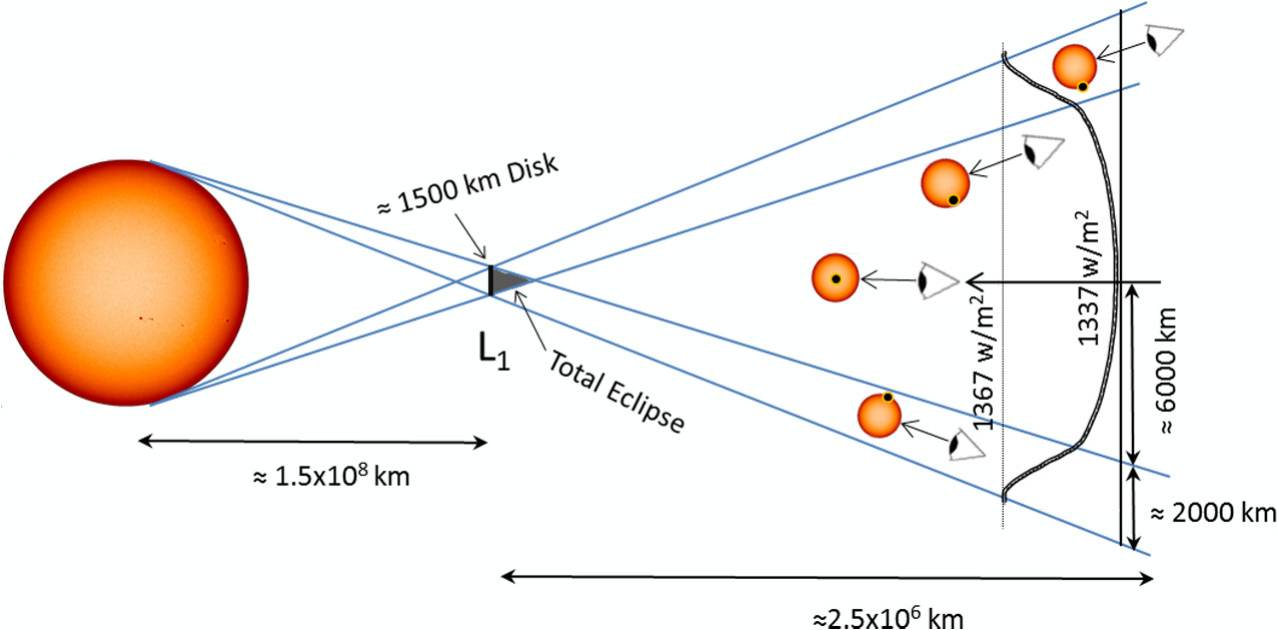
Schematic of the occulting disk shade as seen from the Earth as a function of distance to the Sun-Earth line.

The scenario considering a step-change doubling of the CO_2_ concentration, represented in Fig 4, successfully reproduces the main large-scale features of global warming, with a stronger warming over land (Fig 4*A*), a polar winter amplification (Fig 4*B*) and a stronger warming on the Northern hemisphere (in both Fig 4*A* and Fig 4*B*). As discussed in the original GREB publication [22], after computing the response to the same transient CO_2_ concentration used to compute the IPCC-models [26], some differences can also be noted with the IPCC ensemble mean response (see Figs 5 and 6 from the original paper [22]). The most notable difference is over the arctic winter where GREB does not warm as much as the IPCC models. Also, regions with strong ice-albedo feedbacks tend to warm too much in GREB (i.e. Himalayas). Despite these differences, as argued in [22], the GREB response to global warming is within the uncertainty of the IPCC ensemble.

Hence, GREB is regarded as a fast and flexible tool that provides us with sufficient accuracy of the sensitivity of the climate to the shade cast by the occulting disks in order to gain new insights into the potential of future space-based geoengineering proposals. Nevertheless, more precise analysis will require more accurate climate models (e.g., EMICs or CGCMs), further discussion on the optimal features of a geoengineering climate (i.e. rain patterns, ice cover, extreme events, etc), but also higher fidelity dynamical models for the motion of the occulting disks to account for the perturbations of the Moon and other planets.

### Sun-Earth L_1_ Occulting Disk

GREB may be first used to update the size estimate for a single circular sunshade at the optimal distance from the Earth (2.44x10^6^ km), which in Section I was computed by means of the simple relation in Eq 1 assuming the classical (i.e. no SRP) L_1_ point. The shade cast by the occulting disk is computed and the resultant solar insolation pattern is used as an input to GREB. By means of an iterative secant method, the required radius of the occulting disk can be adjusted in order to obtain a climate response with a global annual-mean surface temperature of 14.1 C°, the same as GREB computed for the control scenario: a CO_2_ concentration of 340 ppm (1xCO_2_) with no artificial shading. The result of this process shows that a 1,434 km radius disk is required in order to negate the effects of GHG emissions globally. Fig 5 shows both the daily-averaged latitudinal incoming solar radiation over a complete year and the shade cast by a 1,434 km radius disk on the Earth. Note that the large increase of size from the previous estimate (i.e. 915 km according to Section I) is mainly due to the shift sunwards of the equilibrium position due to the SRP.

The incoming solar radiation and shade cast by the sunshade are computed here assuming that the Earth moves on a circular orbit around the Sun with a radius of 1 Astronomical Unit and with a constant axial tilt of 23.4 degrees so receiving a solar flux S = 1367 W/m^2^. A disk of 1,434 km radius located at a distance of 2.44x10^6^ km from the Earth would shade the Earth as described in Fig 5*B*. As noted earlier, the shading pattern is computed considering solar limb darkening, the effect that the limb of the solar disk appears darker than the disk centre. The Eddington approximation for solar limb darkening is used here as a reasonable approximation of this effect for the accuracy required. Hence, as described in [27], the ratio of intensity to that of the centre of the disk is given by:

$$\frac{I(\theta )}{I(0)}=\frac{2}{5}+\frac{3}{5}\cos\left(\theta \right)$$ (9)

where *θ* is the angle between the direction of the Sun’s centre and the observer. For an observer located far from the disk, it is a reasonable approximation to substitute cos(*θ*) by $\sqrt{1-u^{2}}$, where *u* is the normalised radial distance to the centre of the solar disk, thus *u* is 1 at the edge of the disk.

Fig 6 represents the observed reduction of the solar flux due to the occulting disk shade as the distance to the Sun-Earth line increases. The figure represents a schematic of the solar flux intensity seen from an observer at the Earth if the occulting disk was located at the SRP-displaced Sun-Earth L_1_ point, albeit the scale has been modified for clarity. Thus, for example, in a location on the Earth where the Sun is in its zenith, i.e. in the central axis in the figure, the solar flux would be 1,337 w/m^2^ at the top of the atmosphere for an occulting disk of 1,434 km radius, while as the observer moves away from the Sun-Earth line, and the Sun moves away from the observer’s zenith, the intensity of the shade decreases in accordance with Eddington’s approximation.

#### Climate Response

**Fig 7:**
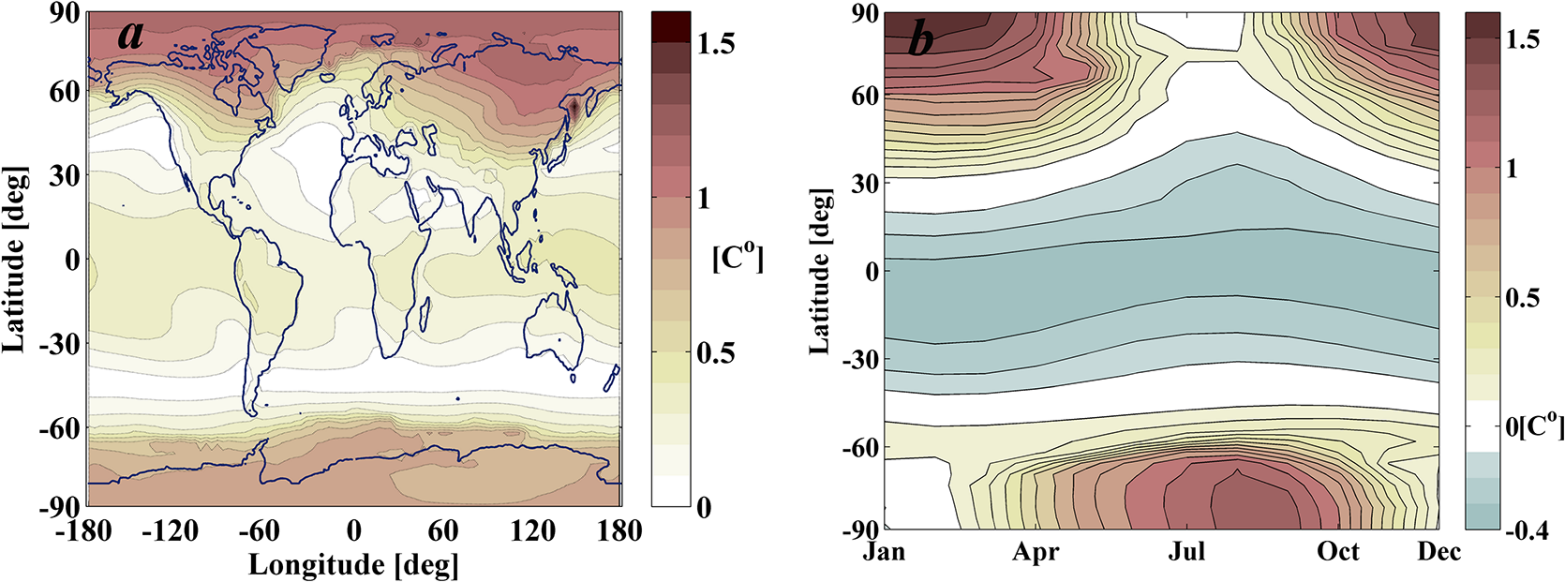
2xCO_2_ geoengineered world scenario as computed by GREB model in a 50 year simulation. The scenario assumes a circular disk of 1,434 km radius placed on the Sun-Earth line at a distance from Earth of 2.44x10^6^ km. *a*) Root-mean-square^1^ (rms) differences of monthly mean surface temperatures between the geoengineering and control scenarios for the year 50 of the simulation. *b*) Latitudinal and seasonal variations of temperature between the geoengineering and control scenarios (averaged in longitude).

The climate response to the shade cast by a 1,434 km radius disk on a 2xCO_2_ world is shown in Fig 7. In particular, Fig 7*A* and 7*B* show the differences in surface temperature between the scenario considering a 2xCO_2_ (680 ppm) geoengineered world and the control scenario (i.e. 1xCO_2_ world (340 ppm) and no artificial shading). Fig 7*A* shows the root mean square (*rms*) differences of monthly mean surface temperatures between the geoengineering and control scenarios for the last year of the simulation, after the climate has reached equilibrium. The *rms* is preferred here, over the annual average, to avoid excessive smoothing when averaging over seasonal variation of temperatures. Fig 7*B* instead shows latitudinal and seasonal variations of the geoengineered climate by averaging over longitudinal surface temperature (i.e. the information on the temperature sign is kept). It is clear from Fig 7 that, despite the fact that a 1,434 km occulting disk cancels almost perfectly the global change of surface temperature, the regional and seasonal effects are still notable. In fact, the classical space-based geoengineering scenario of placing a sunshade at the L_1_ point only manages to maintain less than 10% of the Earth’s surface within 0.1 C° of the surface temperatures of the 1xCO_2_ world (only the white coloured regions in Fig 7*A*). Finally, it is also worth noting that GREB successfully reproduces the same large-scale regional effects of a ‘sunshade world’ as predicted in GCM by Lunt et al. [17] (see Fig 2*A* in Lunt et al.), particularly the warming at the poles and cooling at sub-tropical latitudes.

### Geoengineering Performance Index

In the remainder of the paper, optimal sunshade configurations will be sought that return the largest possible fraction of Earth’s surface to a climate within ±0.1 C° difference of the surface temperatures of that of the control scenario (1xCO_2_). This will be attempted by performing the minimization of a geoengineering performance index that considers the root-mean-square (*rms*) difference of temperature with respect to the control scenario and averages this over the entire Earth’s surface. Importantly, the use of *rms* ensures that the optimization process will not converge towards solutions where excessive cooling during one season is used to compensate for warming in another. Whenever the root-mean-square temperature of a latitudinal node is smaller than or equal to 0.1 C°, the node will account as having a negligible temperature difference with respect to the control scenario. The purpose of the latter is to ensure that the optimization process seeks solutions that bring as many nodes as possible within an acceptable range of temperatures of ±0.1 C° difference with respect to the control scenario.

The Earth’s surface temperature response to a given climate scenario is computed by GREB in a 3D matrix such as *T*_*scenario*_ (*φ*,*λ*,*t*), where monthly mean surface temperatures are stored for each node in the horizontal surface grid (i.e. latitude *φ* and longitude *λ*) and time *t* (in monthly time steps). The matrix $T_{scenario}^{\bar{\lambda }}\left(\varphi ,t\right)$,

$$T_{scenario}^{\bar{\lambda }}\left(\varphi ,t\right)=\frac{\sum_{j=1:96}T_{scenario}^{}\left(\varphi ,\lambda_{j},t\right)}{96}$$ (10)

retains information on the climate’s latitudal and seasonal effects by averaging over the longitudinal grid. The subscript *scenario* denotes the particular setup up of the simulation.

By subtracting $T_{1xCO_{2}}^{\bar{\lambda }}\left(\varphi ,t\right)$ from the control scenario to $T_{GeoEng:2xCO_{2}}^{\bar{\lambda }}\left(\varphi ,t\right)$ of the geoengineering scenario considered, the latitudinal and seasonal effects of the geoengineering scenario are brought forward (e.g., Fig 7*B*):

$$\Delta T\left(\varphi ,t\right)=T_{GeoEng:2xCO_{2}}^{\bar{\lambda }}\left(\varphi ,t\right)-T_{1xCO_{2}}^{\bar{\lambda }}\left(\varphi ,t\right)$$ (11)

The performance index *J* is finally computed by calculating the *rms* over the last 12 monthly mean surface temperatures (i.e. 50^th^ year).

$$\begin{array}{l} \Delta T_{scenario}^{AnnualRMS}\left(\varphi \right)=\\ \sqrt{\frac{1}{12}\left(\Delta T{\left(\varphi ,t_{50^{th}\;year}^{Jan}\right)}^{2}+\Delta T{\left(\varphi ,t_{50^{th}\;year}^{Feb}\right)}^{2}+\ldots +\Delta T{\left(\varphi ,t_{50^{th}\;year}^{Dec}\right)}^{2}\right)} \end{array}$$ (12)

$\Delta T_{scenario}^{AnnualRMS}$ is then a latitude vector of 48 different annual *rms* seasonal variations of temperature. Thus, in order to compute a single performance index the following summation is performed:

$$\begin{array}{l} \Delta T_{f}\left(\varphi_{i}\right)=\{\begin{array}{c} \Delta T_{scenario}^{AnnualRMS}\left(\varphi_{i}\right)\;\text{if}\;\Delta T_{scenario}^{AnnualRMS}\;\left(\varphi_{i}\right)>0.1\left[C^{o}\right]\\ 0\;\text{if}\;\Delta T_{scenario}^{AnnualRMS}\;\left(\varphi_{i}\right)\leq 0.1\left[C^{o}\right] \end{array}\\ J=\sum_{i=1:48}w\left(\varphi_{i}\right)\Delta T_{f}\;\left(\varphi_{i}\right)\\ w\left(\varphi_{i}\right)=\frac{\cos\left(\varphi_{i}\right)}{\sum_{i=1:48}\cos\left(\varphi_{i}\right)} \end{array}$$ (13)

Note first that *w*(*φ*_*i*_) is the weight of the latitudinal area, since the total perimeter of a latitude band is 2*πR*_⊕_ cos(*φ*) where *R*_⊕_ is the radius of the Earth. Hence by summing up *w*(*φ*_*i*_)Δ*T*_*f*_(*φ*_*i*_) for all the latitudinal nodes, we obtain the *rms* change of temperature over the entire Earth’s surface. Clearly, in a rigorous sense, the minimisation of Eq 13 is not the same as the maximisation of a surface area within a given temperature difference, however, as it is argued below, this expression allows a similar result while showing also other advantageous features.

Eq 13 was defined after much heuristic trial an error, in which the following was observed: If the geoengineering performance focused on maximising the surface area within a given temperature difference, then the regions outside these areas were totally neglected by the optimizer, and, as a consequence, the temperature differences in those regions were much larger than otherwise necessary. On the other hand, if no ‘negligible temperature change’ threshold was defined, the consequence was that the optimizer was putting a lot of effort in obtaining a band in the mid-latitudes with an extremely small temperature change, while allowing temperature differences on the order of several tenths of a degree in most of the Earth’s surface. It was thus deemed that a threshold of ±0.1 C°, as negligible temperature difference, was adequate to avoid the optimizer to strive after the minimization of temperature differences below a limit that cannot possibly be resolved by GREB, considering the accuracies of the model. For all of this, it was opted here for a geoengineering performance index such as Eq 13, in order to attempt to maximize the surface area within ‘negligible warming’, while keeping under control the rest of the Earth’s surface.

Many other performance indexes have been proposed in the literature in an attempt to provide quantitative measurements of the anthropogenic damage to the Earth’s environment. For example, as the environmental risk and damage to the Earth are believed to be proportional to a nonlinear function of changes in surface temperature [18], some authors use quadratic functions in temperature change [19]. Also, different weightings for the horizontal grid differences have been proposed, such as by population density or by economic output [28]. In this paper we have, however, chosen a simpler approach [28], where each region is only weighted in terms of its surface area *w*(*φ*_*i*_), and thus the paper will attempt to minimize the climate change impact over the entire Earth’s surface.

## Results and Discussion

### Optimal Configuration for Sun-Earth L_1_ Occulting Disk

**Fig 8:**
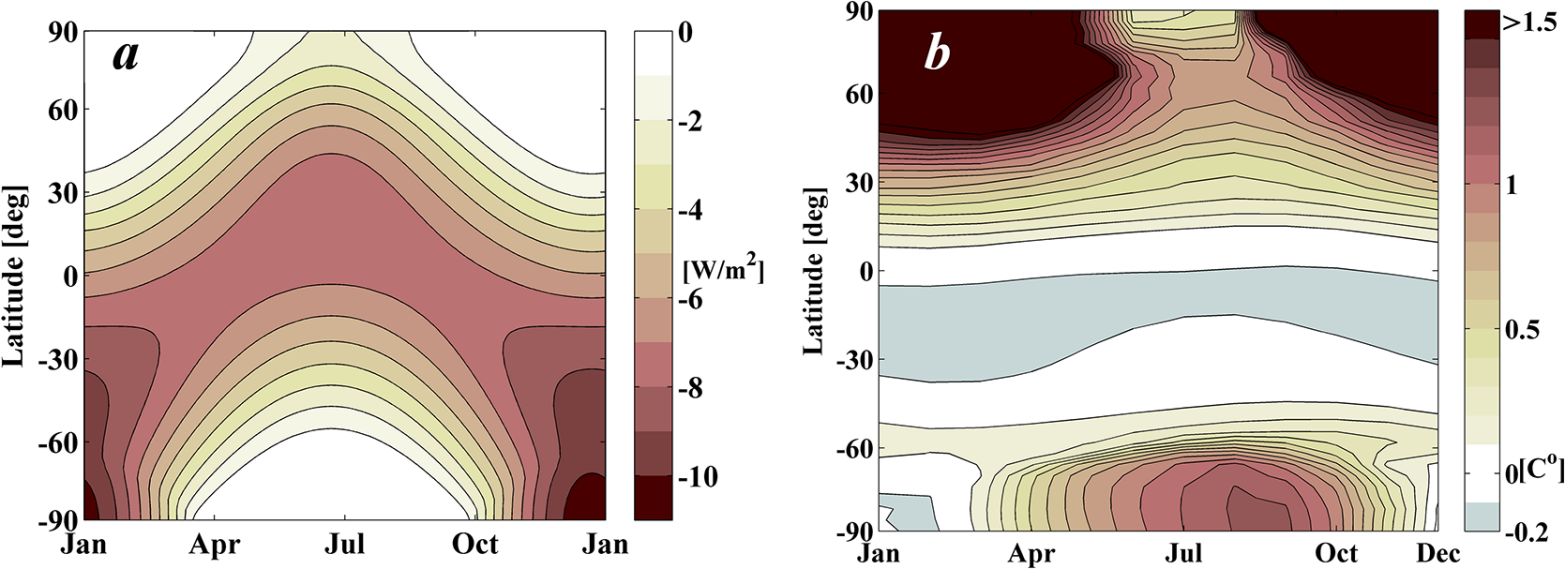
Shade pattern and climate response for displaced occulting disks. *a*) Daily-averaged latitudinal shade over a complete year. Shade cast by a 1,434 km radius occulting disk located at a distance from the Earth of 2.44x10^6^km and displaced by 6,000 km below the plane of the Sun-Earth system. *b*) Climate response for the shade pattern. Climate response to shade as in *a*) in a 2xCO_2_ scenario as computed by GREB model in year 50 of the simulation.

It is clearly understood that by placing the occulting disk in-line with the Sun and the Earth, the maximum amount of shade is cast onto the Earth. However, by displacing the occulting disk from the Sun-Earth line, different shading patterns can be achieved that may affect the global climate differently. Fig 8*A* shows the shade cast by a 1,434 km radius disk but located, instead of in the Sun-Earth line as in Fig 5*B*, at an alternative position displaced by 6,000 km below the plane of the Sun-Earth system. The amount of solar radiation intercepted by the displaced occulting disk is smaller than that of the case shown in Fig 5*B*. Indeed, while for the in-line case the occulting disk intercepted approximately 1.9% of the solar radiation arriving at the Earth, the case shown in Fig 8*A* intercepts 1.5%. Nevertheless, as shown by Fig 8*B*, the latitudinal and seasonal temperature differences in the south hemisphere relax into a state closer to those of the 1xCO_2_ scenario than in the case in Fig 7, albeit the climate impact in northern hemisphere is now clearly worse.

With the results shown in Fig 8, one may envisage that by wasting a small fraction of the shade cast by an occulting disk, it is perhaps possible to find more suitable disk configurations that can reduce the impact of anthropogenic climate change further than that delivered by the simple Sun-Earth in-line configuration. However, these configurations may also require two independent disks such that each occulting disk may tackle more efficiently the changes of temperature at one hemisphere.

A continuum of stationary solutions displaced from the L_1_ equilibrium position exist with specific combinations of sunshade attitudes and lightness parameter *β* [29]. For example, by allowing a tilt to the occulting disk structure of only 0.25° and given the areal mass density required at the minimum mass point at 2.44x10^6^ km from the Earth, out-of-plane displacements of more than 10,000 km could be easily achieved. Note that this tilt is so small that the cross-sectional area seen from Earth varies only by a factor of 10^–5^, which can be considered, for the accuracy intended in this paper, a negligible change. However, not only can stationary configurations for the occulting disks be envisaged, but also certain types of motion near the Sun-Earth L_1_ point may be allowed, as suggested by the existence of families of periodic orbits in the vicinity of the collinear equilibrium points [30]. Allowing for a mobile occulting disk may enable engineered seasonal variations of insolation change across Earth’s surface, and thus allow more efficient control of both latitudinal and seasonal variations of temperature.

With these considerations in mind, we now define a multiple-objective optimization problem that seeks the Pareto optimal design solutions that minimize both the geoengineering performance index *J* and the total shading area required for the two independent sunshades. Unlike single-objective problems, multi-objective optimization seeks a set of feasible solutions of the design variables such that, for each solution of the set, there is no other possible combination of design variables that renders every objective at least as well off, while at least one objective is strictly better off. Consequently, a so-called Pareto optimal solution of the design variables cannot be improved upon without impairing at least one of the objectives.

The two occulting disks are allowed sinusoidal out-of-plane motions *z*(*t*) centred at any displaced position, thus of the type:

z(t)=asin(t+c)+b (14)

where *a*, *b* and *c* are constants to be designed for each sunshade and *t* is an adimensionalized time such that a period of one year is equivalent to 2π. Hence, the general design vector is defined as:

$$x=\left[\begin{array}{ccc} A_{1} & a_{1} & \begin{array}{ccc} b_{1} & c_{1} & \begin{array}{cc} A_{2} & \begin{array}{ccc} a_{2} & b_{2} & c_{2} \end{array} \end{array} \end{array} \end{array}\right]$$ (15)

where *A*_1_ and *A*_2_ are the shading areas of the two mirrors. While the multi-objective criteria vector is defined as:

$$f=\left[\begin{array}{cc} A_{1}+A_{2} & J \end{array}\right]$$ (16)

A controlled elitist genetic algorithm available within MATLAB’s optimization toolbox, *gamultiobj*, was used to construct the Pareto optimal set for the criteria in Eq 16. This algorithm is based on well-established non-dominated sorting genetic programming for multi-objective optimization problems [31]. Unfortunately, constructing such a Pareto optimal set, with any standard multi-objective optimization algorithm, requires a large number of function evaluations of the design variables ***x***. Even with a relatively fast climate model such as GREB, evaluating the climate response to a given configuration of disks size and out-of-plane motions takes on the order of one hour. Therefore, a comprehensive exploration of possibilities, which would require of order 10^5^ evaluations, would require a number of years on a standard single core computer. Fortunately, as noted in previous publications [19, 21], the climate response to a combination of different radiative forcing patterns is very similar to that of the linear combination of climate responses due to each of these radiative forcing patterns.

Taking advantage of this linearity, a set of 1464 different scenarios was computed. Each scenario corresponded to the climate response to an occulting disk of 1,434 km radius located at a distance of 2.44x10^6^ km from Earth, but with a stationary out-of-plane displacement that ranged from 15,000 km above the plane of the Sun-Earth system to the symmetric negative displacement with a step-size of 500 km (i.e. 61 different out-of-plane displacements). For each stationary displacement, 24 different scenarios were considered on which the occulting disk was assumed to be deployed only for half a month at a time, hence no shade was cast during the remaining 11.5 months. Finally, by sampling and combining the climate responses of the set of scenarios, a first estimate of the GREB response to a given shade pattern could be quickly computed. For example, the climate response to the classical space-based geoengineering scenario (i.e. Fig 7) only required summing up the climate responses of the 24 cases corresponding to a zero out-of-plane displacement. Any given motion, defined by Eq 14, could also be computed by summing up all the grid points that most closely matched a given predefined motion *z*(*t*). Eclipses between the motions of the two mirrors were not considered.

**Fig 9:**
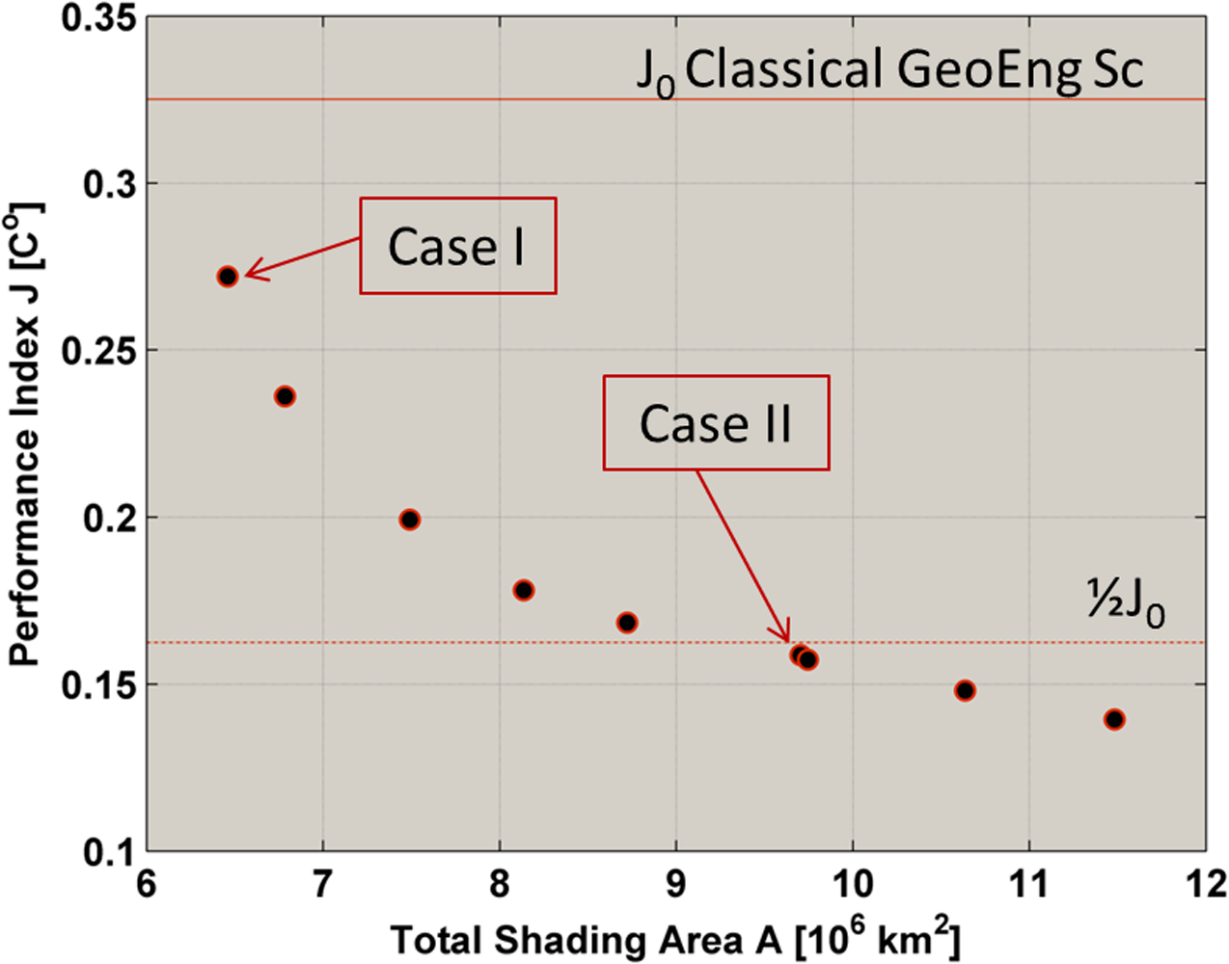
Pareto sets for differently sized occulting disk configurations.

As noted in the previous paragraph, this grid of scenarios was computed using only one single shading area corresponding to the surface area of a disk of 1,434 km radius. When computing a different sized disk of shading area A, a factor A/A_0_ was multiplied to the changes of temperature resulting from the combination of scenarios, where A_0_ is the shading area of the 1,434 km radius disk. Discrepancies due to a nonlinear response to different shading areas were noted between the climate responses of the linear combinations of scenarios and the actual response of the GREB model to the same scenario. Due to this, each solution was then locally re-optimized, using global search genetic algorithms but within a more constrained search domain, with the full GREB model simulations. The final results of this procedure are shown in Fig 9.

Fig 9 shows the Pareto optimal design solutions of the multi-objective criteria defined in Eq 16. The figure also shows two horizontal lines that correspond to the geoengineering performance indexes of the classical Sun-Earth inline solution *J*_0_ and half that value, respectively. The following subsections discuss in more detail two particular solutions within the Pareto set: labelled as case I and II in Fig 9.

#### Case I: Shading area as in the classical in-line configuration

**Fig 10:**
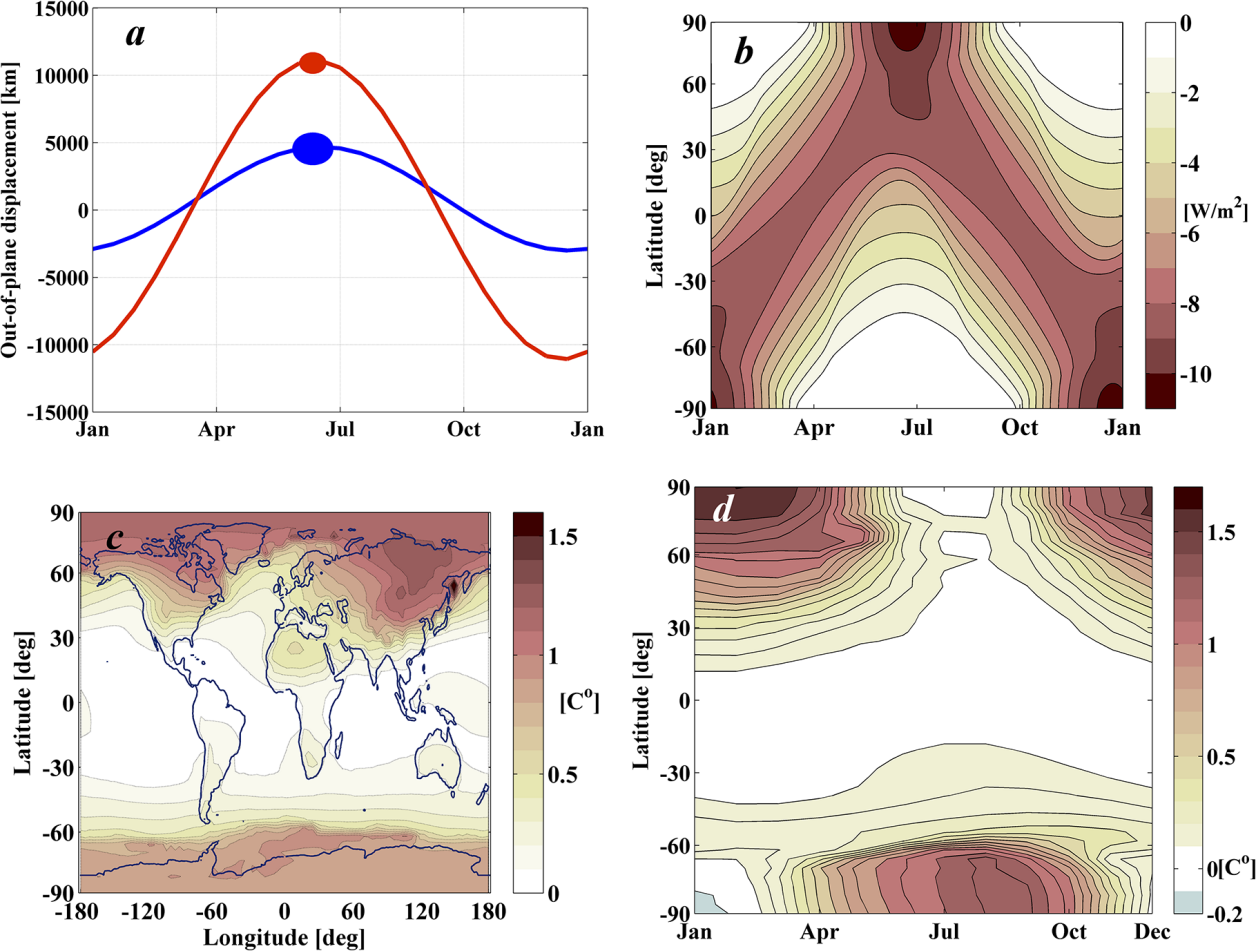
Case I Pareto solution. *a*) Out-of-plane motion of the configuration of two mobile occulting disks. The size of each disk is also represented and scaled with the *z*-axis. *b*) Daily-averaged latitudinal shade over a complete year cast by Case I solution. *c* & *d*) Geoengineered climate response as computed by GREB. *c*) *rms* differences of monthly mean surface temperatures between the geoengineering and control scenarios for year 50 of the simulation. *d*) Latitudinal and seasonal variations of temperature between the geoengineering and control scenarios (averaged in longitude).

The top horizontal line in Fig 9 denotes the performance index achieved by a 1,434 km radius disk in the classical in-line configuration. The Case I solution requires exactly the same total shading area as the classical in-line configuration, i.e. 6.5x10^6^ km^2^, but instead achieves slightly lower levels of latitudinal and seasonal climatic variation. Case I requires two occulting disks of radius 1,200 km and 790 km performing a similarly phased sinusoidal oscillation as shown in Fig 10*A*. Both disks reach their maximum positive displacement at the beginning of the summer in the northern hemisphere. Fig 10*B* describes the shade cast onto the Earth by this configuration of occulting disks, while Fig 10*C* and 10*D* show the climate response as computed by GREB.

The improvement of the geoengineering performance index *J* between Case I and the classical configuration is approximately of 0.05°C. This may seem a small change, but is a noticeable improvement over the climate impacts achieved with the classical geoengineering scenario. This is perhaps more evident by comparing the total area of the Earth’s surface that remains within a 0.1°C of the 1xCO_2_ climate in the Case I solution (Fig 10*C*) with that of the classical geoengineering scenario (Fig 7*A*). Case I returns nearly 40% of the Earth surface to pre-global warming temperatures, while the classical geoengineering scenario achieves less than 10%. Hence, this actually represents a substantial improvement towards the adjustment of the Earth’s climate with exactly the same shading area used in previously published solutions.

#### Case II: Minimum shading area to achieve half J_0_

**Fig 11:**
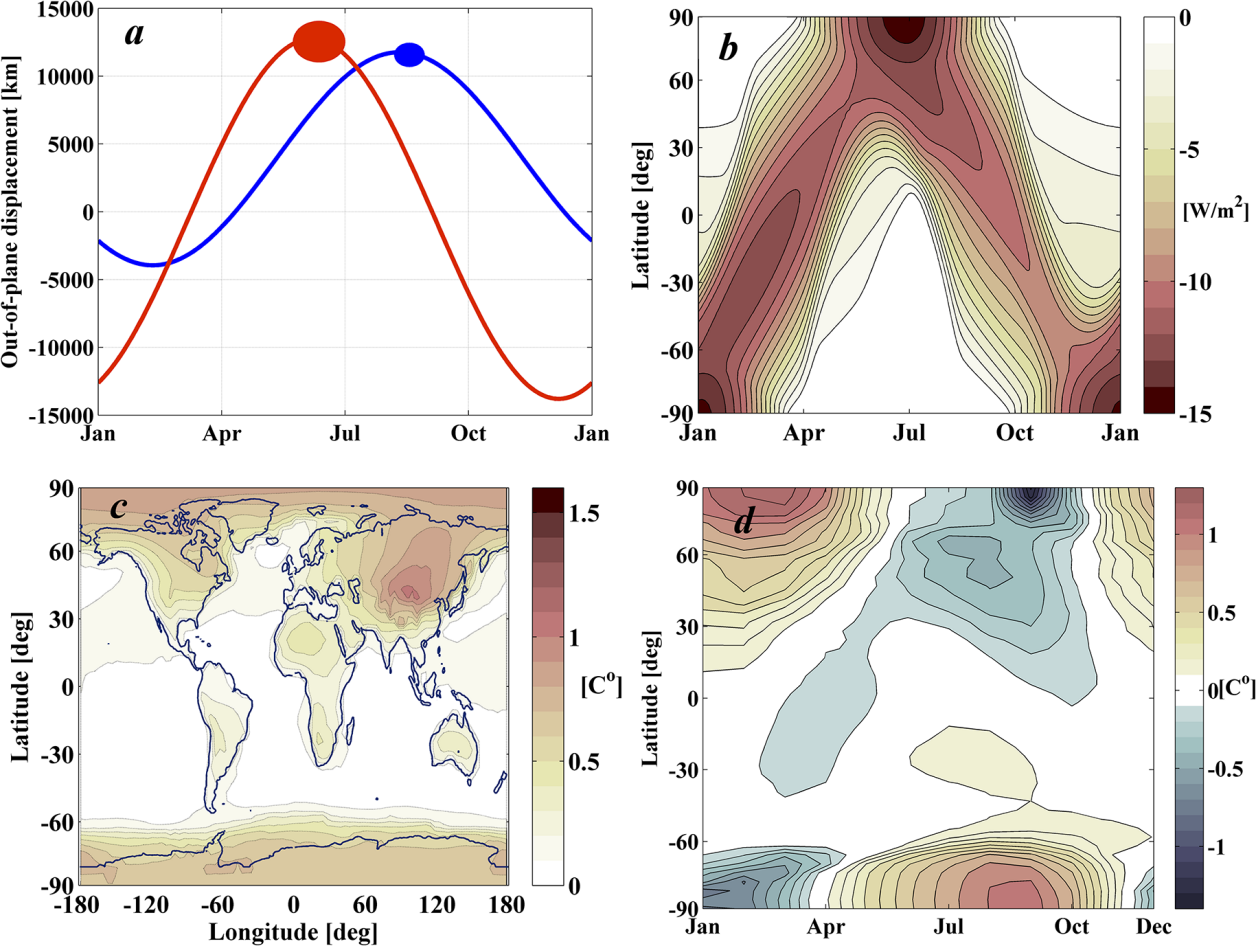
Case II Pareto solution. *a*) Out-of-plane motion of the configuration of two mobile occulting disks. The size of each disk is also represented and scaled with the *z*-axis. *b*) Daily-averaged latitudinal shade over a complete year cast by Case II solution. *c* & *d*) Geoengineered climate response as computed by GREB. *c*) *rms* differences of monthly mean surface temperatures between the geoengineering and control scenarios for year 50 of the simulation. *d*) Latitudinal and seasonal variations of temperature between the geoengineering and control scenarios.

Fig 9 offers a large range of shading areas and performance indices. It can be noted however that the rate at which the performance index decreases with increasing shading area slows as shading area increases. Aside from the Case I solution then, another interesting solution in the set is that requiring the minimum shading area to reduce by half the residual increase of temperatures. This is achieved with the Case II solution, which is described in Fig 11.

Case II requires 1.5 times the shading area of the classical geoengineering scenario, but achieves a geoengineering performance index *J* that is half the performance index achieved by the classical geoengineering scenario. Note that if the environmental risk and damage to Earth is assumed as a quadratic function of the temperature increase [19], that would imply instead that the environmental risk is reduced to a quarter of its original value with an increase of shading area of only 50%. As shown in Fig 11*A*, the two disks necessary for this solution are of 1,522 km and 880 km radius. Note from Fig 11*D* that Case II yields an excessive cooling during polar summer months, nevertheless, as shown in Fig 11*C*, this is still the scenario that reduces most significantly the temperature differences with respect to the control scenario.

### Semi-Analytic Computation of Geoengineering Orbital Configurations

The artificial motion described in Fig 10*A* and Fig 11*A* cannot, however, be achieved either with the stationary solutions described in [29], or with the natural periodic orbits existing in the vicinity of the collinear libration points [30]. Note that the natural out-of-plane periodic motion near the L_1_ is of sinusoidal nature [30, 32], and thus the motion sought benefits from the natural dynamics in the vicinity of the L_1_ point. However, the periodicity of the natural out-of-plane motion does not exactly match that of the one year periodicity required for geoengineering orbits [32], neither the out-of-plane displacements (i.e. *b* parameter in Eq 14) are of natural occurrence. Hence, a specific control law is required in order to ensure that the occulting disk follows the prescribed path. Periodic orbits such that each occulting disk in the configuration satisfies the required out-of-plane motion (i.e. Fig 10*A* and Fig 11*A*) are now sought.

The motion of the sunshades near the SRP-displaced equilibrium points can be conveniently approximated by a first order approximation of dynamics in Eq 4. This approximation is of particular relevance here since the sunshade movement cannot extend further than 15,000 km from the equilibrium position or else the disks would not cast any shade onto the Earth. Let us then move the centre of the reference frame into the position of the SRP-displaced L_1_ point (*x*_e_,0,0) and truncate a Taylor expansion of the equations of motion at first order [32]. The resulting set of differential equations can be written as:

$$\{\begin{array}{c} \ddot{x}-2\dot{y}-\left(1+2c\right)x=a_{x}\\ \ddot{y}+2\dot{x}+\left(c-1\right)y=a_{y}\\ \ddot{z}+cz=a_{z} \end{array}$$ (17)

where the constant *c* = *μ*/(1 − *μ* − *x*_*e*_)^3^ + (1 − *μ*)/(*μ* + *x*_*e*_)^3^ and the sail acceleration vector is now a differential acceleration with respect to that required to obtain an equilibrium position at (*x*_e_,0,0). Thus,

$$\begin{array}{l} a_{x}=\beta \left(1-\mu \right)\left(\frac{1}{{\left(1-\gamma \right)}_{}^{2}}-\frac{1}{r_{1}^{2}}\cos^{3}\alpha \right)\\ a_{y}=\beta \frac{1-\mu }{r_{1}^{2}}\cos^{2}\alpha \sin\alpha \cos\delta \\ a_{z}=\beta \frac{1-\mu }{r_{1}^{2}}\cos^{2}\alpha \sin\alpha \sin\delta \end{array}$$ (18)

where *γ* = *x*_*e*_ − (1 − *μ*). The main advantage of the above description is that the out-of-plane motion is now decoupled from the motion within the Earth’s orbital plane. This allows a simpler implementation of the inverse method to find the control history of the sunshade by inverting the equations of motion.

As described in section I in Results and Discussion, the outcome of the geoengineering optimizations is an out-of-plane motion of the type *z*(*t*) = *a* sin(*t* + *c*) + *b*. Thus, we then know that the sail acceleration in the out-of-plane direction must be such that:

$$\begin{array}{l} a_{z}=\ddot{z}\left(t\right)+cz\left(t\right)\\ z\left(t\right)=a\sin\left(t+c\right)+b\\ \ddot{z}\left(t\right)=-a\sin\left(t+c\right) \end{array}$$ (19)

We may also require that the sunshade does not move away from the Sun-Earth line, i.e. *y* = 0, so that the shade is always cast symmetrically over the morning and afternoon side of the Earth. Thus,

$$a_{y}=2\dot{x}$$ (20)

Precisely because of the two previous constraints, the control history of the clock angle *δ* must then satisfy:

$$\delta =\arctan\left(\frac{\ddot{z}\left(t\right)+cz\left(t\right)}{2\dot{x}}\right)$$ (21)

On the other hand, the control history of the cone angle *α* must also satisfy the following expression:

$$\cos^{2}\alpha \sin\alpha =\frac{r_{1}^{2}\left(\ddot{z}\left(t\right)+cz\left(t\right)\right)}{\beta \left(1-\mu \right)\sin\delta }$$ (22)

If Eqs 21 and 22 are satisfied, the shape of the motion in the *z* and *y* coordinates will be as prescribed, and only *x*(*t*) remains to be solved.

The last constraint imposed on the geoengineering orbit is that it must be periodic, and thus that at the end of one complete revolution its position and velocity must be the same as at the beginning. To satisfy the latter condition, we must then solve a boundary value problem and search for a set of initial conditions $\left[\begin{array}{cc} x_{0} & {\dot{x}}_{0} \end{array}\right]$ such that after one complete Earth period *T*_⊕_, the condition $x_{0}-\phi^{T_{⊕}}\left(x_{0}\right)=0$ is also satisfied, where *ϕ* ^Δ*t*^ (**x**_0_) denotes the flux of the system propagated by a timespan Δ*t*, which in this case is defined by the differential equation:

$$\ddot{x}-\left(1+2c\right)x=a_{x}$$ (23)

**Fig 12:**
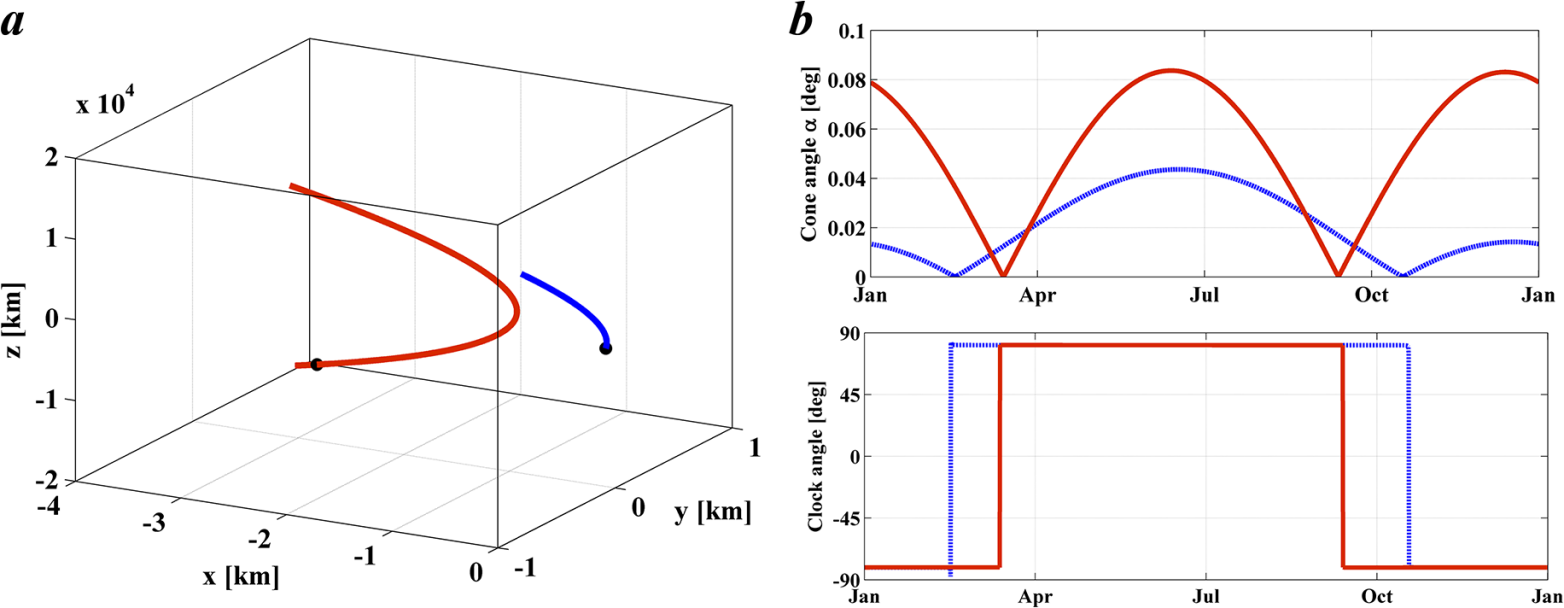
Case I geoengineering periodic orbit. Artificial geoengineering configuration of two occulting disks with out-of-plane motion that satisfy *z*(*t*) = 3,850·sin(*t*-0.423π)+840 [km] (blue orbit) and *z*(*t*) = 11,100·sin(*t*-0.4π)+17 [km] (red orbit). *a*) 1 year period motion in the Earth rotating reference frame, centred on the SRP displaced L_1_ point. *b*) Cone and clock angle (α, δ) control law required to generate the required orbit.

These constraints are met with an artificial orbit for Case I such as that shown in Fig 12*A*, where the Cartesian coordinates have origin at the SRP-displaced L_1_ Lagrange point with the *x*-axis along the Sun-Earth line, *z* normal to the Sun-Earth line and *y* completing the triad. Fig 12*A* represents two periodic orbits with the *y* component constrained as *y*(*t*) = 0 and *z*(*t*) = 3,850 sin (*t* − 0.423*π*) + 840 [km] (blue orbit) and *z*(*t*) = 11,100 sin (*t* − 0.4*π*) + 17 [km] (red). Initial conditions and control laws were then found that allowed the required orbit to be followed by small changes in the orientation of the occulting disk. The control laws are shown in Fig 12*B* defined by the cone angle *α* and clock angle *δ* as a function of time [23].

**Fig 13:**
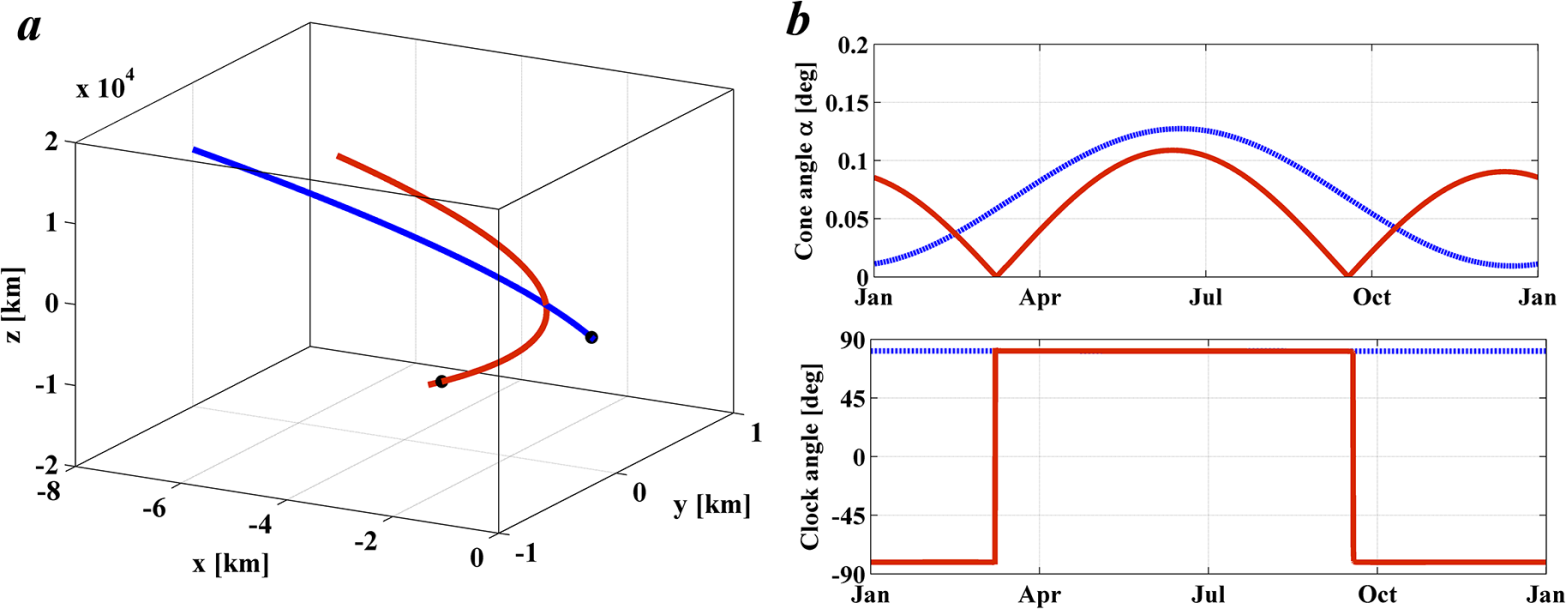
Case II geoengineering periodic orbit. Artificial geoengineering configuration of two occulting disks with out-of-plane motion that satisfy *z*(*t*) = 7,856·sin(*t*-0.72π)+3,908 [km] (blue orbit) and *z*(*t*) = 13,272·sin(*t*-0.36π)-526 [km] (red orbit). *a*) 1 year period motion in the Earth rotating reference frame, centred on the SRP displaced L_1_ point. *b*) Cone and clock angle (α, δ) control law required to generate the required orbit.

Again, for Case II, Fig 13*A* represents two periodic orbits with the *y* component constrained as *y*(*t*) = 0 and *z*(*t*) = 7,856 sin (*t* − 0.72*π*) + 3,908 [km] (blue orbit) and *z*(*t*) = 13,272 sin (*t* − 0.36*π*)-526 [km] (red). Fig 13*B* show the control laws that allow the prescribed motion. Note that in this case the two orbits cross each other, which clearly implies a collisional risk for the two occulting disks. This however can be easily avoided by allowing a small quantity of additional mass (i.e. ballast) in the smallest of the two occulting disks so that its SRP-displaced equilibrium position is displaced by small amount. For example, a mass increase of up to 0.1% would shift the SRP-displaced equilibrium by up to 1,000 km Earthwards. This displacement would affect negligibly the shading patterns calculated here, and thus the validity of the solution.

It is important to note that the geoengineering periodic orbits described here are largely enabled by the underlying natural motion existing in the vicinity to the equilibrium point. This can be noted firstly because the motion strongly resembles that of the naturally occurring vertical motion [30]. This however was expected, since the motion prescribed by Eq 14 matches very closely that occurring naturally. However, the naturally occurring vertical motion has a 2π/*c* period, and this is just slightly short of one year. Hence, the attitude of the occulting disk should be such that the out-of-plane motion is slowed down. The latter can be observed in Figs 12 and 13, particularly by looking at the clock angle law. A small cone angle α and a clock angle δ near 90° means a small tilt looking ‘downward’, and therefore the impingement of photons generates an upward force. Note that this configuration always occurs when the occulting disk is above the ecliptic plane. However, as shown by Eq 19, above the ecliptic, the occulting disk should have a negative acceleration, yet the attitude is such that the SRP provides a positive acceleration, hence effectively slowing down its natural motion.

### Further Remarks

This paper provides new insights into the possibilities offered by space-based geoengineering using orbiting solar reflectors: nevertheless, it does not address practicality issues such as the design and deployment of the sunshades. As noted earlier, the deployment of sunshades in space has been shown to be one of the most effective SRM techniques in terms of the potential cooling achieved with a given change in radiative forcing [13], with comparable efficiency to that of stratospheric aerosol schemes. However, the latter is generally considered as one of the most affordable and timely techniques, while sunshades are not [2]. The excessive cost of space transportation and the high number of launches required to deploy even the ultra-lightweight refractive screens proposed by Angel [12] (with a mass-to-area ratio of about 4 g/m^2^) are seen as the main shortcomings.

Potentially disrupting technological advances such as that of space resource utilization may bring significant leverage to space-based geoengineering schemes [25]. Similarly, there are also synergies to be exploited between space solar power and sun-shade climate control. The occulting structures investigated here aim at some form of dissipation, refraction or reflection of the solar energy that would otherwise intercept the Earth. If this energy was converted instead to laser or microwave and beamed to Earth, as envisaged by solar power satellites [33], the sunshades could also be supplying clean power [34], which would greatly increase the benefits of the scheme. Indeed, it can be speculated that such schemes could be economically self-sustaining [35].

The scale of the geoengineering scheme studied here is clearly vast, and requires a leap of the imagination over current space engineering endeavours. It has been argued nevertheless that the cost of such an endeavour will still be comparable to other terrestrial geoengineering proposals [2]. In terms of human engineering projects, the mass and scale of the sunshades will also be similar to current terrestrial civil engineering projects such as the Chinese Three Gorges Dam [20], and require a mass production of coated thin material equivalent to the current world decadal production of aluminium foil. Nevertheless, scholarly work has yet to identify a scientific showstopper for its implementation [36].

This work represents a multidisciplinary effort to explore the potential for future active control of insolation by means of multiple sunshades flying in formation near the L_1_ point. These configurations were designed with two orbiting occulting disks, but the use of a larger number of disks may also be possible, adding further degrees of freedom to the insolation control. The clime model used provided only a strongly simplified account of the climate processes. Nevertheless, the model reproduces with rough accuracy both the main large-scale regional aspects of the warming patterns [26], and the ‘sunshade world’ geoengineered response [17]. Clearly, more accurate climate models are necessary to validate the resulting climate responses. However, the accuracy of the current models is sufficient to conclude that an out-of-plane sinusoidal motion would likely mitigate some of the large-scale regional temperature differences yielded by a static sunshade deployed at L_1_ point [17]. Also, that this motion is actually enabled by underlying natural dynamics in the vicinity of the L_1_ point, and that the sunshade control laws only attempt to slow down the already naturally occurring motion so that it matches that of the one year period necessary for geoengineering purposes. Hence, while more accurate climate models are required to conclude the extent of the regional mitigation achieved by the control of the insolation, the configuration computed with more advance models are likely to benefit from the same underlying dynamics highlighted here. Finally, it was also discussed the fact that the definition of the performance index has a strong influence over the optimizer response. This is a clear indication of the ability of the sunshades to yield different shading patterns, with accordingly different climate responses.

## Conclusions

The paper has revisited the space-based geoengineering concept of deploying large orbiting sunshades to counteract anthropogenic climate change. For the first time, optimal configurations of orbiting sunshades were investigated that not only offset a global temperature increase, but also mitigate regional differences such as latitudinal and seasonal difference of monthly mean surface temperature. Different configurations of two orbiting occulting disks were presented that achieve clear gains with respect to a static disk near the Sun-Earth L_1_ point. It is acknowledged, however, that the sunshade configurations elaborated here may need to be validated with more complex climate models. Nevertheless, the paper demonstrated that once a series of indices are defined that quantitatively measure how well a geoengineering climate matches an ideal target climate, more optimal configurations for space sunshades can be found than the classical static deployment of a sunshade at the L_1_ point.

## Supporting Information

S1 File The supporting file contains the five Matlab scripts described below. In order to run any of these scripts, the folder *ToolBox PLOSOne SI* must be in the Matlab path. *SI1plot_PlodOneFig9*: The MATLAB script *SI1plot_PlodOneFig9*.*m* loads the data pack *ParetoDataPLOSOne*.*mat* and reproduces figure 9 from PLOS one paper. The data pack (i.e., *ParetoDataPLOSOne*.*mat*) contains the raw data produced by the multiobjective optimization as described in the paper. *SI2plot_PLOSOneFig10_CASEI*: The MATLAB script *SI2plot_PLOSOneFig10_CASEI*.*m* loads the data pack *ParetoDataPLOSOne*.*mat* and reproduces all figures presented in Fig 10: CASE I Geoengineering Solution. *SI3plot_PLOSOneFig11_CASEII*: The MATLAB script *SI3plot_PLOSOneFig11_CASEII*.*m* loads the data pack *ParetoDataPLOSOne*.*mat* and reproduces all figures presented in Fig 11: CASE II Geoengineering Solution. *SI4plotOrbit_PLOSOne_CASEI*: The MATLAB script *SI4plotOrbit_PLOSOne_CASEI*.*m* generates the artificial geoengineering orbits that satisfy the out-of-plane motion required for CASE I solutions. The computation follows the description in Section II of Results and Discussion. *SI5plotOrbit_PLOSOne_CASEI*: The MATLAB script *SI5plotOrbit_PLOSOne_CASEI*.*m* generates the artificial geoengineering orbits that satisfy the out-of-plane motion required for CASE II solutions. The computation follows the description in Section II of Results and Discussion.
(ZIP)
